# Retraction: Chrysin attenuates myocardial ischemia–reperfusion injury by inhibiting myocardial inflammation

**DOI:** 10.1039/d1ra90006d

**Published:** 2021-01-20

**Authors:** Laura Fisher

**Affiliations:** Royal Society of Chemistry Thomas Graham House, Science Park, Milton Road Cambridge CB4 0WF UK advances-rsc@rsc.org

## Abstract

Retraction of ‘Chrysin attenuates myocardial ischemia–reperfusion injury by inhibiting myocardial inflammation’ by Jingguo Wu *et al.*, 2018, **8**, 13739–13746, DOI: 10.1039/C8RA00590G.

The Royal Society of Chemistry hereby wholly retracts this *RSC Advances* article due to concerns with the reliability of the data. The images in the article were screened by an image integrity expert.

In Fig. 2, the expert found several duplicating features in each of the three micrographs, confirming that these images have been manipulated.

Duplicating features were also observed in the IR/HMGB1 and CH/HMGB1 panels in Fig. 6A, indicating that these images had been manipulated.

The expert also raised concerns with the integrity of the Western blots in Fig. 7 and 8. Analysis of the blots in Fig. 7 revealed uncharacteristic white marks around the bands. Analysis of Fig. 8 showed that the first and third band in the P65 panel are unexpectedly similar, and there are potential splice marks in the control GAPDH panel.

The authors were asked to provide the raw data for this article, but did not respond. Given the significance of the concerns about the validity of the data, and the lack of raw data, the findings presented in this paper are not reliable.

The authors have been informed but have not responded to any correspondence regarding the retraction.

Signed: Laura Fisher, Executive Editor, *RSC Advances*

Date: 7^th^ January 2021

## Supplementary Material

